# Recuperative Hostels

**Published:** 1917-06-16

**Authors:** J. Stewart Mackintosh

**Affiliations:** (Chairman of Original Committee for establishing Recuperative Hostels). The Recuperative Hostels, Fitzjohn's Avenue, N.W.


### THE EDITOR'S LETTER-BOX.

### Recuperative Hostels.

*To the Editor of* THE HOSPITAL.

SIR,—I am so convinced that there is a need for the recuperative hostels, in which Sir Frederick Milner has taken so much interest, that I feel it my duty to ask you to allow us to remove a wrong impression created by an article which appeared in your issue of the 19th ult., and has been written, I feel sure, under a misapprehension as to the real objects of these hostels, for they are not intended for the treatment of insane people, as is suggested in your article, but for men who are sane, but who have the misfortune to be suffering from the effects of shell-shock and overstrain of the nervous system. Without the help that the hostels can give them, these men, after their discharge from the Army, must either become a burden to their relatives, and without the means of obtaining the special treatment they require, or they must be content to remain in pauper asylums under surroundings that cannot be regarded as the most favourable to their recovery.—Yours faithfully,

J. STEWART MACKINTOSH, M.D.
(Chairman of original Committee for establishing Recuperative Hostels).

The Recuperative Hostels, Fitzjohn's Avenue, N.W., June 5, 1917.

